# Graduate medical education-led continuous assessment of burnout and learning environments to improve residents’ wellbeing

**DOI:** 10.1186/s12909-022-03366-y

**Published:** 2022-04-18

**Authors:** Dotun Ogunyemi, Ali Ghassan Darwish, Gregory Young, Erica Cyr, Carol Lee, Sarkis Arabian, Kedar Challakere, Tommy Lee, Shirley Wong, Niren Raval

**Affiliations:** 1 Arrowhead Regional Medical Center, Colton, CA USA; 2 California University of Science & Medicine, Colton, CA USA

## Abstract

**Background:**

Promoting residents’ wellbeing and decreasing burnout is a focus of Graduate Medical Education (GME). A supportive clinical learning environment is required to optimize residents’ wellness and learning.

**Objective:**

To determine if longitudinal assessments of burnout and learning environment as perceived by residents combined with applying continuous quality Model for Improvement and serial Plan, Do, Study, Act (PDSA) cycles to test interventions would improve residents’ burnout.

**Methods:**

From November 2017 to January 2020, 271 GME residents in internal medicine, general surgery, psychiatry, emergency medicine, family medicine and obstetrics and gynecology, were assessed over five cycles by Maslach Burnout Inventory (MBI), and by clinical learning environment factors (which included personal/social relationships, self-defined burnout, program burnout support, program back-up support, clinical supervision by faculty, and sleep difficulties). The results of the MBI and clinical learning environment factors were observed and analyzed to determine and develop indicated Institutional and individual program interventions using a Plan, Do, Study, Act process with each of the five cycles.

**Results:**

The response rate was 78.34%. MBI parameters for all GME residents improved over time but were not statistically significant. Residents’ positive perception of the clinical supervision by faculty was significantly and independently associated with improved MBI scores, while residents’ self-defined burnout; and impaired personal relations perceptions were independently significantly associated with adverse MBI scores on liner regression. For all GME, significant improvements improved over time in residents’ perception of impaired personal relationships (*p* < 0.001), self-defined burnout (*p* = 0.013), program burn-out support (*p* = 0.002) and program back-up support (*p* = 0.028). For the Internal Medicine Residency program, there were statistically significant improvements in all three MBI factors (*p* < 0.001) and in clinical learning environment measures (*p* = 0.006 to < 0.001). Interventions introduced during the PDSA cycles included organization-directed interventions (such as: faculty and administrative leadership recruitment, workflow interventions and residents’ schedule optimization), and individual interventions (such as: selfcare, mentoring and resilience training).

**Conclusion:**

In our study, for all GME residents, clinical learning environment factors in contrast to MBI factors showed significant improvements. Residents’ positive perception of the clinical learning environment was associated with improved burnout measures. Residents in separate programs responded differently with one program reaching significance in all MBI and clinical learning environment factors measured. Continuous wellbeing assessment of all GME residents and introduction of Institutional and individual program interventions was accomplished.

**Supplementary Information:**

The online version contains supplementary material available at 10.1186/s12909-022-03366-y.

## Introduction

Burnout has been described as a prolonged response to chronic emotional and interpersonal stressors on the job defined by three dimensions: emotional exhaustion, depersonalization, and a sense of reduced personal accomplishment [1, 2]. Accreditation Council for Graduate Medical Education (ACGME), Common Program Requirements emphasizes that programs, in partnership with their Sponsoring Institutions, have the same responsibility to address burnout and improve well-being as they do to evaluate other aspects of residents’ competence. Programs must ensure healthy and safe learning and working environments that promote resident well-being [3].

It has been suggested that the origins of burnout among residents are rooted in the learning environment. Vendeloo et al. found strong and consistent inverse association between the perceived quality of the learning environment and burnout among residents in a Dutch national study. They concluded that the learning environment is of key importance in preventing resident burnout [4]. A study from Thailand on pediatric residents also demonstrated a positive relation between educational climate and work-related quality of life [5]. The clinical learning environment independent of personal characteristics was found to be associated with residents' burnout in Iran [6].

It is well documented that after entering training, residents’ burnout rates increase significantly compared to their peers [1, 6–8]. A meta-analysis that included 22,778 residents reported that the prevalence of burnout in several Asian countries was 57.18%; in several European countries it was 27.72% and in North America it was 51.64%. 7 Furthermore, it is important to not only measure burnout but to also introduce effective mitigating interventions that can improve the learning environment and resident’s wellness. Interventions to prevent burnout may be more effective when focused on changing the system rather than individual physicians [9]. Actionable guidelines for residency programs to mitigate burnout and to promote wellbeing have been published [8–11]. Parson et al. in a narrative summary identified best practice recommendations for individual interventions which were 1) mindfulness training, 2) resilience training programs, 3) encouraging self-care and 4) faculty mentoring and support of at-risk residents. For institutional interventions, the best practice recommendations were 1) wellness committee; 2) dedicated institutional resources, 3) wellness curricula, 4) workflow interventions and 5) avoidance of excessive work hours with residents’ schedule optimization [9]. 

Drybe et al. noted that limited data are available regarding how to best address trainee burnout, but multi-pronged efforts, with attention to culture, the learning and work environment and individual behaviors, are needed to promote trainees’ wellness and to help those in distress [12]. Individual residency programs have reported on outcomes of wellbeing interventions [13–16]. However, there are fewer reports on institutional Graduate Medical Education (GME) system-wide intervention wellbeing programs [1, 17]. Even more important are the need for longitudinal sustainable burnout prevention and wellness promotion programs over time using continuous quality improvement and Plan Do Study Act (PDSA cycle). PDSA cycles are the building blocks of continuous improvement and shares commonalities with other cyclical frameworks such as Kolb's learning cycle or the Knowledge to Action cycle that are familiar to medical educators. The use of PDSA echoes the scientific method in that it looks for failure, adjusts accordingly and grows knowledge incrementally over time [18].

Therefore, the objective of this study was to determine if longitudinal assessments of Maslach Burnout Inventory (MBI) scores and perceptions of the clinical learning environment by residents at approximately six months intervals as outcome measures over five cycles using principles of improvement science and a Plan Do Study Act process with the introduction of institutional and individual program interventions would be associated with improvements in the outcome measures. Our hypothesis was that using the Model for Improvement and serial Plan, Do, Study, Act cycles to test interventions would improve residents’ burnout.

## Methods

This is a report on a continuous quality improvement model utilized by GME to improve burnout and wellbeing perceptions of residents by burnout and program clinical learning environment surveys obtained from residents using time-series evaluation at 5 points from November 2017 to January 2020. Using a PDSA process, at each time point, residents’ survey results were analyzed by the GME analyst and Designated Institutional Officer (DIO), the data was reviewed with program directors, and interventions implemented by program or GME administrators as indicated.

We used the PDSA continuous quality framework construct. The first step in the cycle is the Plan step which involves identifying an aim or purpose, formulating a theory, assembling the quality improvement team, defining process, outcome or balancing metrics, determining changes or interventions to be tested and putting a plan into action. This is followed by Do step in which the plan and interventions or changes are implemented, and relevant data are collected. In the Study step, the team monitors and reviews the outcomes to test the validity of the plan and interventions for signs of progress and success, or problems and areas for improvement. The Act step closes the cycle, integrating the new knowledge generated by the process, which can be used to adjust the goal, change methods, reformulate a theory altogether, or broaden the learning-which leads into the next PDSA cycle. These four steps can be repeated over and over as cycles of continual learning and improvement [18].

The GME office at Arrowhead Regional Center includes ACGME accredited programs in Internal Medicine, Emergency Medicine, Family Medicine, Obstetrics & Gynecology, Psychiatry and General Surgery. The GME office initiated the conduction of a bi-annual well-being survey of all residents beginning in the Academic Year 2017/2018. The Mid-Year Well-Being Survey was conducted from November to January, while the End-of-Year Well-Being Survey was conducted from April to May. All residents were surveyed using longitudinal web-based survey instruments. The surveys for this report included the full MBI (22 items); and a program clinical learning environment survey which included sub-sets on: impaired personal/social relationships (3 items), self-defined burnout (1 item), program burnout support (2 items), program back-up support (3 items), clinical educational supervision (4 items), and sleep difficulties (1 item) (Supplementary Information).

The learning environment survey was developed by the GME staff analyst based on a review of the literature and knowledge acquired from working with program directors and residents on wellness. Face validity was conducted by the DIO and program directors who evaluated, reviewed, modified, and finally determined that the surveys tested the intended themes.

The results of each survey were compiled by a GME analyst and reviewed with the Designated Institutional Officer (DIO). The DIO used the wellbeing survey findings in combination with other data such as the ACGME survey results to strategize and implement institution-wide action plan and interventions. The unique data for each residency program was shared and reviewed with the program director and department leadership who likewise used the report in combination with ACGME survey findings as feedback to plan and develop appropriate interventions and strategies. Residents did not see the results of the surveys but participated in discussions, town hall meetings and focus groups but were not involved in executive decisions regarding interventions. The data and proposed interventions were also reviewed, deliberated with decisions made at the program directors meeting and the Graduate Medical Education Committee meetings.

For the study, our outcome measures were: a) MBI items of emotional exhaustion (9 items), depersonalization (5 items), personal accomplishment (8 items); b) program clinical learning environment survey (14 items). Data was displayed in a run chart over time to monitor if implemented interventions or changes were leading to improvements.

Interventions to achieve outcomes that were introduced in the PDSA process included both system wide and program specific interventions. Organization directed interventions that affected workflow and the learning environment that included leadership changes, faculty recruitment, administrative support and technological innovations were coordinated in collaboration with GME. GME advocated and acquired required financial support and resources allocation for program support and needs. GME further coordinated effectively between c-suite, medical staff, residency programs and other ancillary services to achieve desired wellbeing support for residents’ workflows concerns. Interventions that coincided with the desirable MBI trends included the provision of protected time for residents’ healthcare needs during working hours, regular wellness events sponsored by GME for all residents, and a yearlong focused wellness program for interns. Other Interventions involved successive strategies such as focus group meetings with residents with the development of strategies to address concerns. Changes in the operational leadership including Chief Medical Officer, Designated Institutional Officer, a departmental chair, and program directors facilitated a focus on the institutional academic mission, increased faculty, and ancillary staff (nurse practitioners and physician assistants) recruitment and workflow improvements in clinical service teams.

Kruskal–Wallis test was used to determine significant trends over time. Based on the five tests performed for the longitudinal trend, a Bonferroni-corrected alpha level of *p* = 0. 0125 (0.05/4) was reported per test as significant for the Kruskal–Wallis test. Multivariate Analysis of Variance (MANOVA) analysis using General Linear Multivariate Model was used to test the statistical significance of the differences between groups. Linear regression was used to identify statistically significant independent associations. IBM SPSS Statistics for Windows, version 20(IBM Corp., Armonk, N.Y., USA) was used for statistical analysis. The study was approved by the institutional IRB.

## Results

Overall, 271 residents from six residency programs were surveyed five times over three years with 1350 responses on surveys of MBI, and various clinical learning environment factors that could impact residents’ well-being. Residents’ completion of the surveys over the five points survey were: 82% (134/163); 81.6% (133/163); 76.8% (129/168); 78% (131/168) and, 73.3% (124/169) respectively (mean = 78.34%). Responses by specialty were as follows: Family Medicine = 360 (26.7%); Internal Medicine = 315 (23.3%), Emergency Medicine = 245 (18.1%), General Surgery = 180 (13.3%), Psychiatry = 155 (11.5%), and Obstetrics and Gynecology = 95 (7%),

For all GME residents, emotional exhaustion scores decreased over time from 28.12 (categorized as high) to 24.35 (categorized as moderate) which was significant on initial analysis but became non-significant when corrected for multiple analysis. Depersonalization scores decreased from 12.48–10.65 (both categorized as moderate), and personal accomplishment scores increased from 38.51–40.05 (categorized as moderate to high) over time, but the trends did not reach significance (Table 1).

**Table 1:** Maslach Burnout Inventory scores of all GME residents at 5 points analyzed by Kruskal–Wallis test for trends and MANOVA

	December 2017; *n* = 130	May 2018; *n* = 131	December 2018; *n* = 126	May 2019; *n* = 133	December 2019; *n* = 121	Kruskal–Wallis *p* value	MANOVA *p *value
Emotional Exhaustion Score	28.12 (0.93)	26.56(0.91)	24.60 (0.98)	25.31(0.95)	24.35 (0.96)	0.04!	0.03*
Depersonalization Score	12.48 (0.58)	11.65(0.51)	10.60 (0.61)	10.81(0.56)	10.65(0.56)	0.074	0.092
Personal Accomplishment Score	38.51 (0.55)	38.14(0.62)	39.84(0.56)	39.41(0.58)	40.05(0.54)	0.113	0.078

Legend:

() = standard error of the mean

*N *= total number residents who completed the survey for each time period

Results of Kruskal–Wallis test for trends:

! Based on the five tests performed, a Bonferroni-corrected alpha level of *p* = 0. 0125 (0.05/4) was reported per test as significant for the Kruskal–Wallis test. Thus, the *p* value of 0.04 was not significant in the Kruskal–Wallis test for trends

Results of MANOVA analysis:

*There was homogeneity of variance-covariances matrices, as assessed by Box’s test of equality of covariance matrices (*p* = 0.705). Wilks’ Lambda multivariate statistics *p* value was 0.205 which showed that there were no significant differences between the groups. On tests of between-subjects effects Emotional Exhaustion Score had *p* value of 0.03 which became non-significant *p* = 0.055 on post-hoc Bonferroni correction

**Fig. 1:**
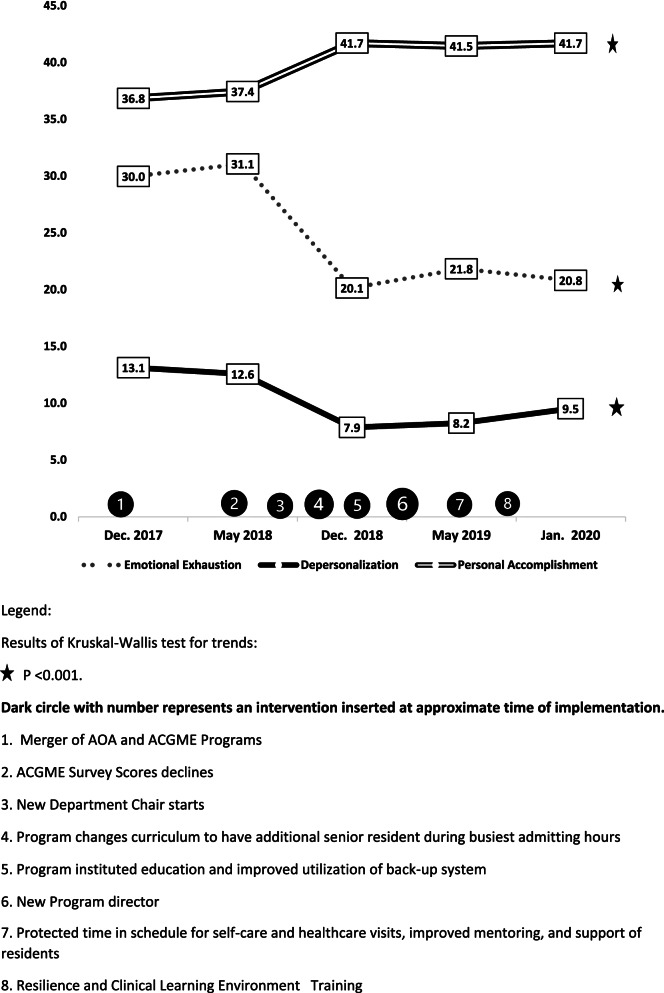
Trends of Maslach Burnout Inventory of residents in the Internal Medicine Residency program surveyed in five cycles from December 2017 to January 2020 analyzed by Kruskal–Wallis test for trends

Kruskal–Wallis test for trends analyses of the MBI scores of each residency training program showed similar trends which reached significance only for the Internal Medicine residency training program. Figure 1 displays the MBI results for the Internal Medicine residency training program. This revealed that emotional exhaustion scores decreased over time from 30.12 [categorized as high] to 20.8 [categorized as moderate] (*p* < 0.001). Depersonalization scores decreased from 13.1 [categorized as high] to 9.5 [categorized as moderate] (*p* < 0.001); and personal accomplishment scores increased from 36.8 [categorized as moderate] to 41.7 [categorized as high] over time (*p* < 0.001).

**Fig. 2:**
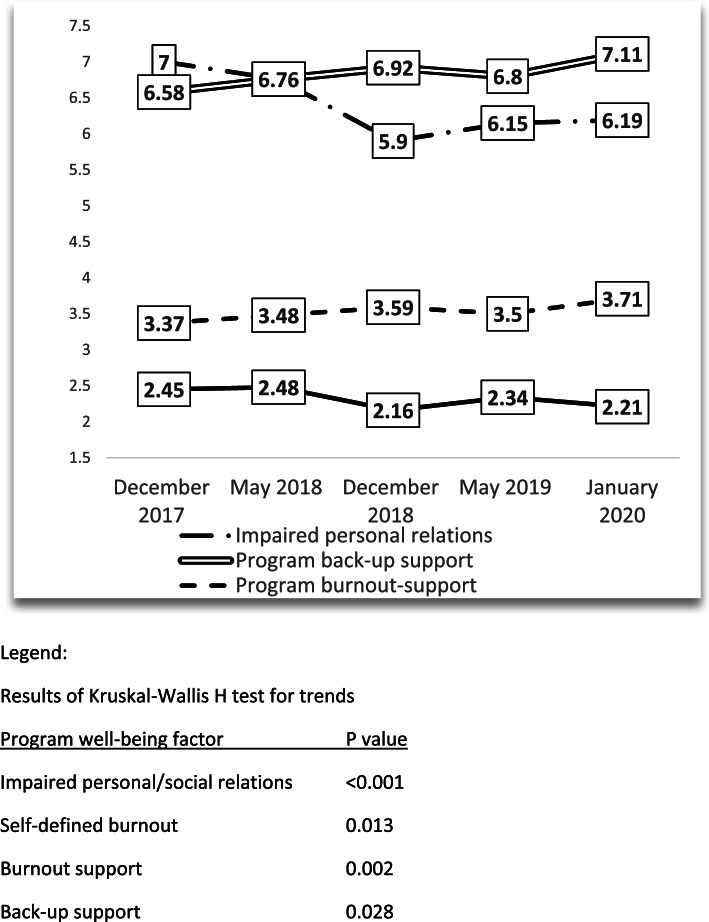
Trends of Clinical Learning environment scores of all GME residents surveyed in 5 cycles from December 2017 to January 2020 analyzed by Kruskal–Wallis test for trends

Figure 2 demonstrates the results of the trends in the residency clinical learning environment factors. Residents’ perception of impaired personal or social relations scores decreased from 7.00 to 6.19 (*p *= 0.001); self-defined burnout scores decreased from 2.45 to 2.21 (*p* = 0.013); residents’ perception of program’s support for burnout increased from 3.37 to 3.71 (*p *= 0.002); residents’ perception of program’s support for back-up scores increased from 6.58 to 7.11 (0.028), clinical educational supervision scores increased from 9.10 to 9.44 (*p* = 0.613) and sleep difficulties decreased from 1.74 to 1.68 (*p* = 0.071).

**Fig. 3:**
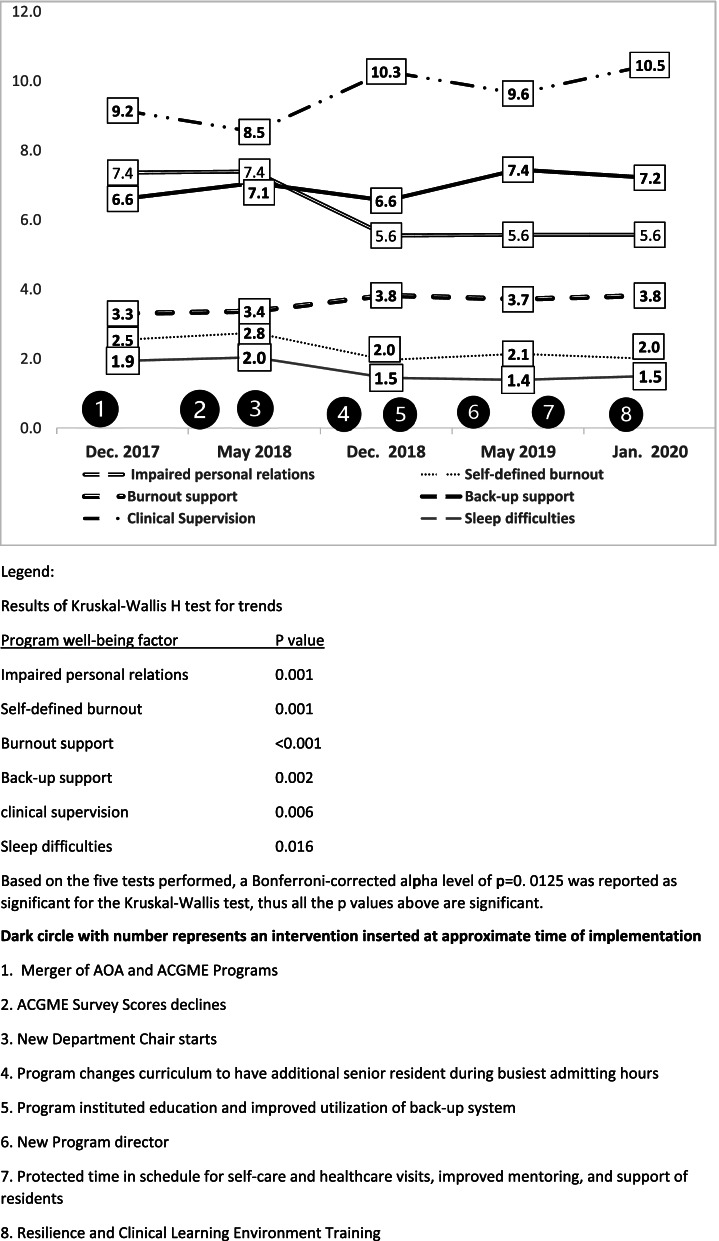
Program learning environment trends of residents in the Internal Medicine Residency program surveyed in 5 cycles from December 2017 to January 2020 analyzed by Kruskal–Wallis test for trends

Analysis of the clinical learning environment of each residency training program showed similar trends which again reached significance for the Internal Medicine residency training program. Figure 3 displays the scores for the Internal Medicine residency training program. Residents’ perception of impaired personal/social relations scores decreased from 7.4 to 5.6 (*p* = 0.001); self-defined burnout scores decreased from 2.8 at the 2nd time-point to 2.0 (*p* = 0.001); residents’ perception of program’s support for burnout increased from 3.3 to 3.8 (*p* < 0.001); residents’ perception of program’s support for back-up scores increased from 6.6 to 7.2 (*p* = 0.002), favorable clinical education supervision scores increased from 9.2 to 10.5 (*p* = 0.006), and sleep difficulties decreased from 1.9 to 2.5 (*p* = 0.016).

On linear regression analysis; self-defined burnout; and impaired personal relations were independently significantly associated with increasing depersonalization and emotional exhaustion scores but with decreasing personal accomplishment scores. Conversely, clinical supervision was independently significantly associated with decreasing depersonalization scores and emotional exhaustion but increasing personal accomplishment (Table 2).

**Table 2:** Linear regression analysis with Depersonalization; Emotional Exhaustion; Personal Accomplishment as dependent variables

Variable	Unstandardized Coefficients: B	Standardized Coefficient: beta	*P* value	95.0% Confidence Interval for B
Depersonalization as the dependent variable				
Clinical supervision	-0.527	-0.164	< 0.001	-0.805 to -0.249
Self-defined burnout	2.019	0.256	< 0.001	1.319 to 2.719
Impaired personal relations	0.597	0.221	< 0.001	0.380 to 0.815
Backup support	-0.741	-0.157	0.001	-1.170 to -0.312
Emotional Exhaustion as the dependent variable				
Clinical supervision	-1.134	-0.214	< 0.001	-1.486 to -0.781
Self-defined burnout	4.660	0.358	< 0.001	3.771 to 5.549
Impaired personal relations	1.308	0.293	< 0.001	1.031 to 1.584
Backup support	-0.624	-0.080	0.025	-1.169 to -0.079
Personal Accomplishment as the dependent variable				
Clinical Supervision	1.052	0.329	< 0.001	0.785 to 1.340
Self-defined burnout	-1.434	-0.183	< 0.001	-2.159 to -0.709

Legend

Variables entered into the models included: Clinical supervision; Self-defined burnout; Impaired personal relations; Back-up support; Burnout-support; Sleep difficulties as independent variables and Emotional Exhaustion, Depersonalization, Personal Accomplishment were the dependent variables

Only significant findings were reported

Figures 1, and 3 list in chronological order, some of the interventions in the Internal Medicine residency program with each PDSA cycles. The improvements in residents’ wellbeing perceptions in this program occurred with the collaborative effort and commitment of many stake holders including C-suite executives, GME administration, department administration, faculty and residents. Initially, there were two separate programs operating under the same department and service, one accredited by the American Osteopathic Association (AOA) and the other accredited by the Accreditation Council for Graduate Medical Education (ACGME). In 2017, the two programs were merged as a part of single accreditation system for residency training in the United States.

After the first PDSA cycle, declining ACGME scores led to a review by the executive and GME leadership. Program leadership and hospital administration met with residents to listen to concerns and to develop strategies. As a result, in 2018, a new departmental chair who was contractually committed to the department was appointed as the indicated intervention. The new departmental chair worked with administration and a new chief medical officer, who was also a member of the department, on providing improved faculty supervision, and restructuring clinical workflows. The program instituted curricular changes, improved didactic education, increased faculty mentoring, support of residents and implemented a back-up system for the residents. In 2019, a new program director was appointed in PDSA cycle 3 who further strengthened the program and provided further support for the residents. In PDSA cycle 4, interventions introduced included residents receiving resilience training which included workshops such as emotional intelligence, imposter syndrome; and training to facilitate an inclusive, equitable and fair clinical environment such as implicit bias, and cultural humility. Residents’ planned social activities were increased and they were provided “wellness days” with protected time during work hours for self-care and healthcare visits (Figs. 1 and 3).

## Discussion

For all GME residents, emotional exhaustion, depersonalization, and personal accomplishment subscale scores improved over time, but the changes were not statistically significant. However, all the three MBI subscale measures improved significantly amongst residents in the Internal Medicine residency training program. We noted a high mean baseline rate of burnout (e.g. a mean emotional exhaustion score of 28.12 categorized as high) which is in concordance with the literature, with recent studies reporting physician burnout rates ranging up to 89% [9, 16, 19–22]. Prior research has demonstrated that burnout rates increased significantly during residency training [23]. Rosen et al. reported in a cohort of internal medicine residents who were followed longitudinally that they had significantly more depersonalization and emotional exhaustion by the end of intern year.25 Our overall GME results showing improvement in the MBI measures over time even though not statistically significant may suggest that we may have stemmed or decreased the natural progression of residents’ burnout to worsen during the time period studied [24, 25].

We used a time series evaluation which allows refining of the intervention and monitoring to determine evidence of effectiveness. Time series evaluation using PDSA cycles offer an advantage over simple before-after studies in that they allow teams to draw inferences about whether improvement occurred, as well as whether the intervention led to the observed improvement. 19 Use of PDSA to improve medical education has been reported. Arnstead et al. used PDSA cycles to improve the frequency of Competence by Design assessment in surgery residents in Canada [26]. Dunbar et al. demonstrated that using PDSA cycles improved physician recognition and reporting of patient safety events [27],

The 22-item MBI regarded as the gold-standard self-reported questionnaire was utilized in our study to measure burnout, but some recent studies have used other measures. For example, Choi et al. used a one-item MBI scale and reported less burnout when measured twice from 2011 to 2013 (2.35 to 2.33, *p* = 0.023) [19]. Mari et al. used the Copenhagen Burnout Inventory (CBI) and noted a clinically meaningful reduction in burnout after 1 year of intervention implementation [15].

The benefits of residency training maybe offset by rigorous educational demands, long working hours, lack of autonomy, work/home/life imbalance and a lack of supportive and nurturing environment. These learning environment factors may have detrimental effects on the mental health of residents and a substantial proportion of residents may experience burnout [19]. Thus, an important component of our study was a focus on the assessment of the unique learning environment of each residency training program and the development of appropriate interventions. For all GME residents, self-defined burnout scores showed a statistically significant decrease over time. Since the MBI was not significant at the GME level, this may suggest that a self-defined burnout assessment may be more discriminant. All GME residents also had a decreased perception of impaired social and personal relationships, with improved perceptions of burnout support by the program. Bird et al., also using a nonproprietary single burnout item and questions regarding burn-out support, learner satisfaction and engagement, demonstrated that a wellness curriculum fostered a sense of togetherness among peers and created an additional support system [25].

Even though this was a GME-wide approach, individual programs responded differently. Internal Medicine residency program achieved statistically significant improvement in all MBI and learning environment measures. This varying result is not unexpected since differing program-specific conditions, engagement, academic status, leadership, and learning environments would influence residents’ perceptions. Other investigators have also noted variations in individual residency program responses to wellness interventions. In contrast to our study in which we used organization level interventions, Aggarwal et al. using an individual intervention introduced a 12-week wellness curriculum to five residency training programs and noted that general surgery residents never implemented the booster sessions due to lack of protected time and change in program leadership, while anesthesiology residents had organizational challenges [16]. It is also possible that the improvements observed in the Internal Medicine program may be due to other factors that were not measured. For example, the significant findings may have been due to a larger sample size but should be noted that responses from Family Medicine were more than those of Internal Medicine residents.

To achieve residents’ wellness perceptions, we provided both organization-directed and individual interventions. This is in agreement with previous reports showing that organization directed interventions in comparison to individual interventions were associated with a meaningful reduction in emotional exhaustion and depersonalization scores [11, 28, 29]. DeChant et al. reviewed studies on 4 unique categories of organization-directed workplace interventions of teamwork, time, transitions, and technology. They concluded that organization-directed workplace interventions that improve processes, optimize electronic medical records (EHRs), reduce clerical burden by the use of scribes, and implement team-based care can lessen physician burnout [30]. In our Internal Medicine residency training program with significantly improved outcomes; the associated major interventions were organization-directed including leadership change, faculty recruitment, resources, and service workflow improvements.

Globally more work is required in mitigating burnout and its sequalae such as depression and suicide amongst post graduate trainees. Naji et al. in a systemic review that spanned the past 2 decades noted a pooled prevalence of burnout involving 31 210 residents from 47 countries and reported that the prevalence of burnout remained unchanged over the past 2 decades. Burnout varied by region, with lowest level in European countries. They concluded that current wellness efforts and policies have not changed the prevalence of burnout worldwide and that future research should focus on understanding systemic factors and leveraging these findings to design interventions to combat burnout [31]. Thus, more studies on interventions similar to our study using continuous quality improvement and PDSA cycles are required worldwide.

### Limitations

There are many limitations to this study. The results are from a single county hospital located in an underserved minority community; thus, the results may not be generalizable to different locations. Since this is an observational study without control groups, the reported changes could be caused by other unobserved factors; thus, the reported associations cannot be interpreted as causative. Specifically, we did not focus on the role of ACGME surveys and citations on interventions and residents’ wellbeing perceptions. Though we used the validated MBI survey, our learning environment surveys were developed locally. There is always a concern of incomplete data and selection bias but the response rate of 73–82% would be classified as a high response rate. Other limitations were that each time point included residents from different graduate years and more time may be required for the interventions to be effective. Another potential limitation of this study is that clustering of data may have occurred since datapoints within training programs may be more similar than between training programs.

## Conclusions

To our knowledge, this is one of the few published reports on longitudinal burnout and learning environment assessments among GME trainees utilizing PDSA cycles. MBI parameters improved over time in all GME but were not statistically significant. Over time, all GME residents showed statistically significant improvements in self-defined burnout, personal/social relationships, and burnout support by the program. One residency program had statistically significantly desirable trends for all 3 MBI subscales and all learning environment measures including clinical education supervision and back up support by the program. This study suggests that GME-wide wellbeing assessments and interventions are feasible, and results may vary between residency training programs.

## Supplementary Information

**Additional file 1.** 

## Data Availability

The datasets generated and/or analyzed during the current study are not publicly available due our institutional policies but are available from the corresponding author; Dotun Ogunyemi on reasonable request.
