# Supplementary material for: Graduate medical education-led continuous assessment of burnout and learning environments to improve residents’ wellbeing
Source: BMC Med Educ. 2022 Apr 18;22:292. doi: 10.1186/s12909-022-03366-y (PMC9016951; doi:10.1186/s12909-022-03366-y)
Supplement: Supplementary file 1 — Additional file 1. [file 12909_2022_3366_MOESM1_ESM.docx]

**Supplementary Information**

**Well-Being Survey**

**Disclaimer:**

**Thank you for participating in the well-being survey. The protection of your identity and personal information is important to us. The survey will only be used to analyze overall population trends and to identify any special segments which may need additional resources. All responses will be aggregated by program to provide critical feedback to your program directors. Your individual answers to the following questions will not be shared. We strongly encourage you to take advantage of well-being resources that are available to you through your insurance, your program, and available in the Well-Being Book of Resources. Your well-being matters to us and, as such, we strive to work with you to help improve your experience here at ARMC.**

**Individual Information**

**Clinical Learning Environment Well-Being Assessment**

**Impaired personal relations:**

1. **During the last year, my job has made it harder for me to nurture existing personal relationships.**
   1. Not true at all
   2. Somewhat true
   3. Very true
   4. Completely true
2. **During the last year, my job has made it harder for me to develop new meaningful personal relationships.**
   1. Not true at all
   2. Somewhat true
   3. Very true
   4. Completely true
3. **During the last year, my job has contributed to conflict in my personal relationship(s).**
   1. Not true at all
   2. Somewhat true
   3. Very true
   4. Completely true

**Self-defined burnout:**

1. **Using my own definition of "burnout", please choose one of the choices the best fits you:**
   1. I enjoy my work. I have no symptoms of burnout.
   2. Occasionally I am under stress, and I don't always have as much energy as I once did, but I don't feel burned out.
   3. I am definitely burning out and have one or more symptoms of burnout, such as physical and emotional exhaustion. The symptoms of burnout that I'm experiencing won't go away. I think about frustrations at work a lot.
   4. I feel completely burned out and often wonder if I can go on. I am at the point where I may need some changes or my need to seek some sort of help.

**Program burnout-support**

1. **If you felt you were burnt out, would you feel comfortable speaking to your peers about your well-being?**
   1. No
   2. Yes
2. **If you felt you were burnt out, would you feel comfortable speaking to an attending, program director, or department chair about your well-being?**
   1. No
   2. Yes

**Program back-up support**

1. **Does your program have a policy/procedure or "backup system" in place to ensure patient care in the event that you are unable to perform your duties due to excessive fatigue, illness, or family emergency?**
   1. No
   2. Yes
2. **Do you feel that you can initiate the "backup system" without suffering negative consequences from your program director or faculty?**
   1. No
   2. Yes
3. **Do you feel that you can initiate the "backup system" without suffering negative consequences from your peers?**
   1. No
   2. Yes
4. **Do you feel that your program is providing you adequate protected time for your studies?**
   1. No
   2. Yes

**Clinical Education Supervision**

1. **My program director recognizes my contributions and clinical work.**
   1. Not true at all
   2. Somewhat true
   3. Very true
   4. Completely true
2. **I am satisfied with the level of autonomy I receive.**
   1. Not true at all
   2. Somewhat true
   3. Very true
   4. Completely true
3. **I am learning at a comfortable rate.**
   1. Not true at all
   2. Somewhat true
   3. Very true
   4. Completely true

**Fatigue**

1. **Sleep difficulties**
2. No
3. Yes

**ARMC utilizes the 22-item version of the Maslach Burnout Inventory in order to allow us as an institution to be able to compare levels of burnout with other institutions nationwide. The Maslach Burnout Inventory (MBI) is recognized as the leading measure of burnout and validated by 35+ years of extensive research. The MBI measures burnout as defined by the World Health Organization (WHO) and it is used in 88% of burnout research publications (Boudreau, Boudreau & Mauthe-Kaddoura, 2015).**

[**https://www.mindgarden.com/184-maslach-burnout-toolkit**](https://www.mindgarden.com/184-maslach-burnout-toolkit)
